# The Processing Space of the Spray-Dried Mannitol-Leucine System for Pulmonary Drug Delivery

**DOI:** 10.3390/pharmaceutics16030398

**Published:** 2024-03-14

**Authors:** Riley T. Schweizer, Mani Ordoubadi, Cody A. Prather, Reinhard Vehring, Kimberly B. Shepard

**Affiliations:** 1 Research & Development, Lonza Group AG, Bend, OR 97703, USA; 2 Department of Chemical, Biological, and Environmental Engineering, Oregon State University, Corvallis, OR 97331, USA; 3 Department of Mechanical Engineering, University of Alberta, Edmonton, AB T6G 2E1, Canadavehring@ualberta.ca (R.V.)

**Keywords:** spray-drying, leucine, mannitol, polymorphism, powder stability, nucleation, particle formation

## Abstract

Designing spray-dried particles for inhalation aims at specific physicochemical properties including a respirable aerodynamic diameter and adequate powder dispersibility. Leucine, an amphiphilic amino acid, has been shown to aid in optimizing bulk powder properties. Mannitol, a model crystalline active and common bulking agent, was co-sprayed with leucine at several excipient ratios, ethanol/water ratios, and spray dryer outlet temperatures in order to experimentally probe the underlying particle formation mechanisms in this binary crystalline system. During the droplet drying of two crystallizing components, the material that nucleates first will preferentially enrich the surface. It is desired to have a completely crystalline leucine shell to improve powder properties, however, mannitol competes with leucine for the surface depending on excipient concentration and manufacturing parameters. The resulting particles were studied initially and at a two-month timepoint via solid state characterization, visual analysis, and particle size analysis in order to detect changes in bulk powder properties. It was determined that, similar to systems where only leucine can crystallize, initial leucine saturation in the formulation dictates powder characteristics.

## 1. Introduction

Spray drying is a particle engineering technique used to manufacture inhalable drug products that have many distinct advantages for patient compliance and disease outcomes [1]. Inhalable drug products are typically used to treat lung-specific diseases such as asthma and chronic obstructive pulmonary disease because of the fast onset of action and targeted delivery to the deep lung. The breath-actuated functionality of typical dry powder inhalers (DPIs) allows for effective drug dosing, regardless of patient lung function, and their portability increases patient compliance [2]. Furthermore, the superior stability of dry powders over liquid formulations makes DPIs an attractive solution for medication stockpiling and use in regions without a reliable cold chain [2]. Finally, inhalable dry powder drug products are non-invasive and propellent-free compared to other products, e.g., injectables or metered dose inhalers [3].

Inhalable drug products must be formulated with lung anatomy in mind [4]. The size-dependent deposition throughout the regions of the lung makes it imperative that an inhalable drug product has adequate aerodynamic properties [5]. To be respirable, an aerosol must have a mass median aerodynamic diameter (MMAD) of approximately 1–5 µm [6]. If the particles are too large, they will impact the back of the throat and subsequently be swallowed. Conversely, particles that are too small may be exhaled [6]. This critical role of particle size also puts emphasis on adequate powder dispersibility [7]. If the powders fail to de-aggregate during drug administration, the agglomerated particles act as a single larger particle, potentially making the drug product non-respirable [7]. To overcome this, a specific class of excipients called dispersibility enhancers have been introduced in respiratory drug formulations. One such excipient is leucine which is known for its ability to decrease particle-particle interactions and therefore aids in particle de-aggregation [8].

The formation of the leucine shell creates void space inside the particle leading to reduced particle density and therefore lower MMAD. The rugosity of the leucine shell reduces the contact area between the particles leading to less particle agglomeration [9]. Thus, in order to obtain the desired functionality of leucine, the formulation and process must be designed in a manner to facilitate early leucine surface-enrichment.

In the formulation of inhalable drug products, leucine has been added alongside active pharmaceutical ingredients (APIs) and other excipients acting as bulking agents [10]. Leucine sprayed with an amorphous API such as budesonide and glass-forming excipients such as trehalose has been studied extensively and found to improve aerosol properties [11,12]. However, pharmaceutical APIs tend to precipitate in different ways; some remain amorphous during spray drying while others crystallize [13]. An API’s crystalline form is often preferred for its improved stability and typically low hygroscopicity. Crystalline APIs may also require dispersibility enhancement for lung delivery, but leucine spray-dried in a formulation with another crystallizing excipient or API has not been studied in detail [10].

**Figure 1:**
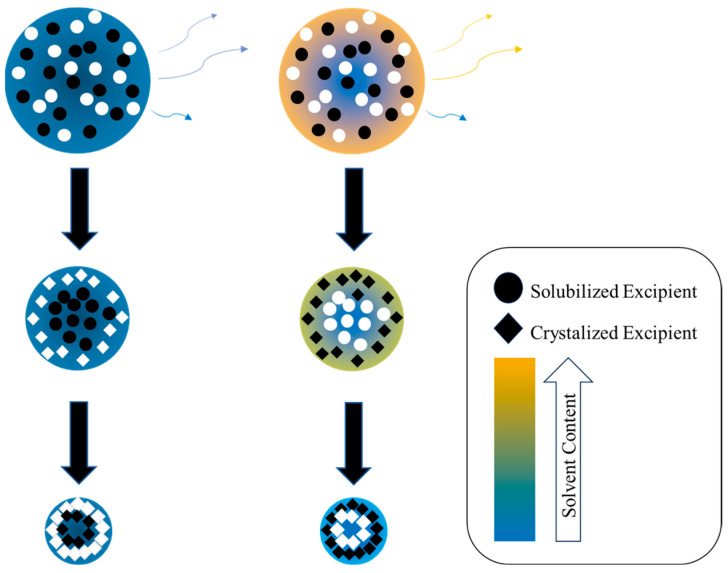
Simplified diagram of single droplet drying and nucleation kinetics for small molecules. It illustrates how changing the solvent blend impacts which excipient supersaturates first and enriches at the surface. In a single solvent system (**left**), given equal initial concentrations, the excipient with lower aqueous solubility (white) crystalizes and enriches the surface first as the solvent evaporates. In a binary system (**right**) in which the solubility of the other excipient is lower, the sequence of nucleation can be reversed (black first then white), which leads to a change in surface composition of the particle.

It is important to be aware of several complexities that arise within a binary crystalline system [14]. For instance, the sequence of nucleation events for the two components during droplet evaporation is dictated by several factors including temperature, excipient concentrations, and solvent composition in co-solvent systems [2]. It has been reported that 90% of new APIs, including those in the respiratory pipeline, are poorly soluble in water [15], and thus require mixed water-organic solvent systems as spray drying solvents. Therefore, a representative co-solvent system is relevant to future research. Leucine is practically insoluble in most organic solvents, slightly soluble in ethanol, and moderately soluble in water at neutral pH with a solubility of ~22 mg/mL [16]. In an ethanol/water system, as a co-solvent droplet dries, the ratio of ethanol to water will generally change due to ethanol’s increased volatility [17]. As depicted in Figure 1, in mixtures with high fractions of ethanol in the solvent mixture, leucine is more likely to nucleate first while an excipient that is more soluble in water will likely nucleate second [18]. If the droplet’s evaporation rate is very high, e.g., due to high dryer temperatures, the droplet may dry very rapidly, leaving little time for crystallization. In this instance, the short crystallization window, i.e., the time available for crystal growth between nucleation and complete evaporation of the solvent, can be insufficient for complete leucine crystallization [17], especially in the presence of other components in the formulation [19]. Thus, adjusting solvent ratios, leucine concentration, and changing drying temperatures play a large role in determining which excipient supersaturates and subsequently nucleates first [20].

To probe the outlined complexities in a binary crystalline system, this study utilized mannitol as a model rapid crystallizer that is relevant for both high- and low-dose DPIs [21]. Mannitol is a non-reducing sugar alcohol that is commonly used in inhalable drug products as a bulking agent and is also approved as an active ingredient for asthma diagnosis at high doses (Bronchitol^®^, Chiesi). Mannitol is especially favorable due to its low hygroscopicity in the crystalline state, leading to desirable stability properties [2]. When studying mannitol’s nucleation kinetics in relation to leucine, it is critical to consider which polymorphic form of mannitol crystallizes [22]. Mannitol is known to have three polymorphic forms: β, α, and δ, in decreasing order of stability [23]. Mixtures of these polymorphs are commonly seen in spray-dried products containing mannitol [24]. Failure of such formulations during stability studies may be related to the inter-conversion between the mannitol polymorphs, which is exacerbated by residual water in the sample due to incomplete drying or unsuitable storage conditions [25]. Understanding the conditions that lead to the nucleation of different polymorphs of an API or excipient is crucial for determining robust manufacturing and storage conditions.

This study aims to provide a foundation for future development work on spray-dried inhalable drug products containing multiple crystalizing components. We studied how leucine interacts with the model crystalizing API, mannitol, during spray drying under differing manufacturing conditions, including excipient ratio, solvent blend, and spray dryer outlet temperature. The effects of each tested parameter were probed by several orthogonal techniques in order to elucidate the processing space of the mannitol/leucine placebo system.

## 2. Materials and Methods

### 2.1. Materials

Materials consisted of L-leucine (Cat. No. A12311.0E, ThermoScientific, Ottawa, ON, Canada), D-mannitol (Cat. No. MA165, Spectrum Chemical, New Brunswick, NJ, USA), and δ-mannitol (Parteck^®^ Delta M, Cat. No. 1.12635, Millipore Sigma, St. Louis, MO, USA). Solvents consisted of DI water from an in-house system and ethanol (Ethyl Alcohol 190 Proof, Cat. No. 111000190, Pharmco, Brookfield, CT, USA).

### 2.2. Methods

#### 2.2.1. Spray Draying

All spray-dried powders were produced on a custom lab-scale dryer preheated to an inlet temperature of 98–170 °C to match a desired outlet temperature (40, 50, 60, or 70 °C). The process was conducted with a nominal nitrogen drying gas flow rate of 500 g/min. Mannitol and leucine were co-dissolved in mixtures of ethanol and deionized water to achieve the desired formulations, all with the same total feed stock concentration of 2 wt.%. Spray solutions were fed to the dryer at a rate of 10 g/min via a peristaltic pump and atomized using a two-fluid nozzle (Model ¼ J, 1650 liquid body and 64 air cap, Spraying Systems Co., Wheaton, IL, USA) at an atomization pressure of 310 kPa. A cyclonic separator with a diameter of 5 cm was used to collect the resulting particles. 

**Figure 2:**
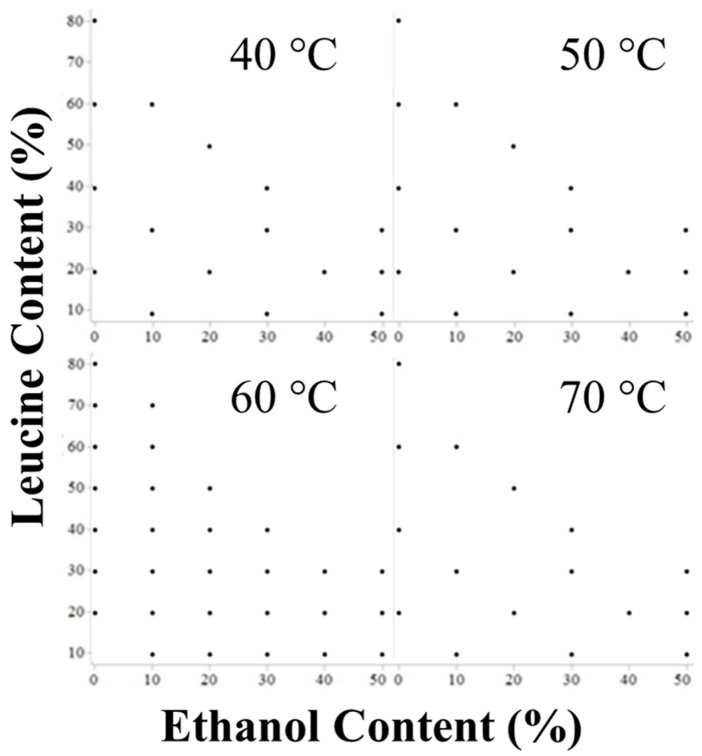
Graphical representation of formulations explored. The x and y axes represent the range of ethanol and leucine concentrations explored, respectively. Formulations are separated by the outlet temperature they were processed at. Each point represents one sample formulation. Note that the 60 °C outlet temperature was more fully explored being a typical operating temperature for aqueous spray drying. All formulations are explicitly listed in Table 1.

Explored formulations are listed in Table 1 and a graphical representation of the formulations explored is shown in Figure 2. Formulations are named as LxxEtxxTxx for the leucine mass fraction (with the balance mannitol), the ethanol solvent mass fraction (with the balance water), and the outlet temperature used for manufacturing. The (c) nomenclature denotes mannitol-only control formulations of the original binary ones.

These formulations cover a broad range of leucine loadings, solvent mixtures, and drying temperatures to fully explore the effects of a crystallizing active, mannitol, in a spray-dried dispersion with a surface-active dispersibility agent, leucine.

#### 2.2.2. Droplet Size Distribution

A droplet size analyzer (SprayTec, Malvern Panalytical, Malvern, UK) equipped with a 4 mW He-Ne laser, 750 mm lens, and a log-spaced array detector was used to determine the initial atomized droplet size distribution of the atomizer. The beam was aimed through the center of the atomization plume once it was fully developed, approximately 10 cm below the orifice. All solvent blends resulted in a spray with a median diameter, *d*_v_(50), of 7–8 µm. The average result, 7.5 µm, was used for the theoretical formulation model. 

#### 2.2.3. Solubility

**Figure 3:**
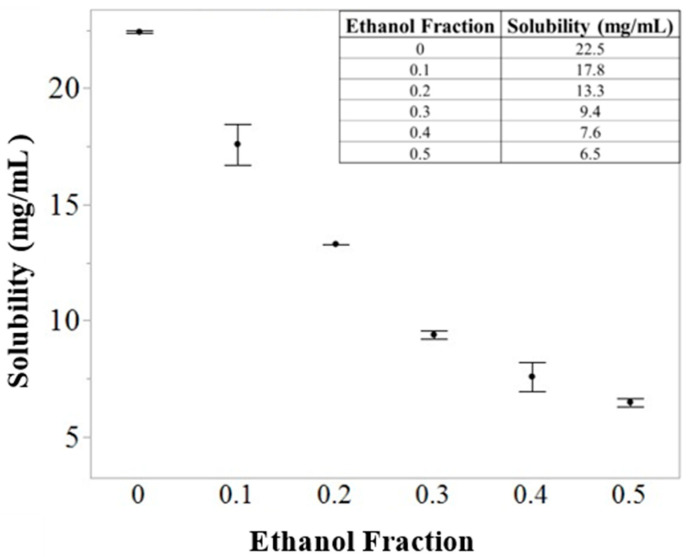
Leucine solubility in ethanol/water mixtures via TGA. Solubility was used to determine experimental space limits.

A thermogravimetric analyzer (TGA) (Discovery Series, TA Inc., New Castle, DE, USA) was used to determine the solubility of leucine in an organic solvent. Solutions with excess leucine were prepared in mixtures of ethanol and water at temperatures of 20–22 °C to mimic the temperature of droplets in the spray dryer due to evaporative cooling [20]. The solutions were then centrifuged, and the saturated supernatant was collected for analysis. A 50 µL sample of each supernatant was dispensed into a tared TGA pan. The TGA pans were allowed to air-dry for 60–90 min. Dried samples were heated at 50 °C/min to 120 °C and held isothermally for 10 min to remove any residual solvent. The solubility of leucine in the ethanol/water mixtures was then calculated using the final sample mass, which can be found in Figure 3.

#### 2.2.4. Theoretical Formulation Model

Mannitol and leucine can both crystallize during spray drying resulting in particle shell formation differences, depending on the sequence of crystallization (see Figure 1). Shell formation is strongly influenced by the time at which a solute reaches critical supersaturation and by its window of crystallization, i.e., the time available for crystal growth until all solvent is evaporated. An advanced particle formation model [12,17] was utilized to correlate experimental outcomes with theoretical critical supersaturation ratios and windows of crystallization in order to explain the morphology of the particles. 

The model is an unsteady numerical solver that progresses in time step-by-step by first estimating the evaporation rates of the solvents in a single droplet, updating the solvent composition, and eventually solving for the spatial distribution of each solute inside the droplet at each step. More detailed information about the exact methodology can be found elsewhere [17]. Due to surface enrichment during droplet evaporation, the maximum concentration of each solute is always on the surface [26]. The instantaneous surface concentrations of both leucine and mannitol were then compared to theoretically obtained critical supersaturation concentrations to estimate the onset of their nucleation and crystal growth [26,27]. A representative series of formulations with experimentally observed morphological differences were modeled as a theoretical comparison. The selected formulations are indicated in Table 1. 

#### 2.2.5. Powder X-ray Diffraction (PXRD)

**Figure 4:**
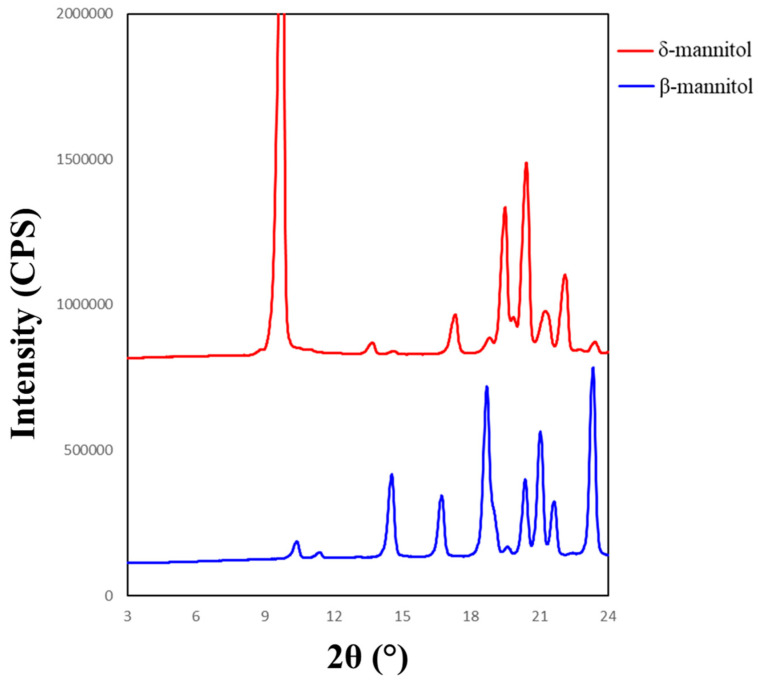
Reference diffractograms for δ-mannitol (**top**) and β-mannitol (**bottom**). Note characteristic peaks at 9.6° for δ-mannitol and 14.6° for β-mannitol.

The initial identification of mannitol polymorphs was performed using an X-ray diffractometer (MiniFlex 600, Rigaku Corporation, Tokyo, Japan). The diffractometer was equipped with a copper anode generator set to 45 kV and 15 mA. Samples were evenly distributed over a 0.2 mm deep zero-background sample holder and gently flattened using a microscope slide. Samples were scanned over a range of 3–40° 2-theta with 2.5 °/min rotational speed. The peak at 9.6° was analyzed for the δ polymorph, the peak at 14.6° was analyzed for the β polymorph, and the peak at 17.2° was analyzed for the α polymorph. Reference diffractograms of β-mannitol and δ-mannitol are shown in Figure 4. The α polymorph was not able to be isolated for characterization. Characteristic peak intensity and broadness was noted for each curve and utilized to qualitatively analyze diffractograms. Quantitative assignment of PXRD peaks was revealed to be unreliable due to the peak tailing from the leucine characteristic peak at 6.2° which caused peak broadening in the characteristic mannitol peaks. 

#### 2.2.6. Aerodynamic Particle Size Distribution

An aerodynamic particle sizer (APS) equipped with an aerosol diluter and powder disperser (Models 3321, 3302 and 3433, TSI, Shoreview, MN, USA) was used to measure the aerodynamic particle size distribution of the powders. The diluter module utilized a 100:1 capillary and was run at a pressure of 80 kPa. The airflow and sheath flow of the powder disperser were set at 18.5 L/min and 4 L/min respectively. The strong dispersive force de-aggregates the powder, which is then measured using time-of-flight calculations. The MMAD was determined by assuming a log-normal particle size distribution. 

#### 2.2.7. Optical Particle Size Distribution

An optical particle size analyzer (Mastersizer 3000, Malvern Panalytical, Malvern, UK) equipped with a powder disperser (Aero S, Malvern Panalytical, Malvern, UK) and log-spaced detector array was used to measure the volume-based equivalent optical particle size distribution of all samples. The inversion algorithm used Mie theory with a refractive index of 1.65 and an absorption coefficient of 0.001. A pressure titration was performed to determine the optimal dispersion pressure. A pressure of 3 bar was utilized to effectively disperse particles without causing particle breakage. 

#### 2.2.8. Scanning Electron Microscopy (SEM)

Particle morphology was determined using a field-emission scanning electron microscope (Model SU3500, Hitachi, Tokyo, Japan). Electron micrographs were captured at an accelerating voltage of 10–15 kV and a working distance of 10–14 mm. Small quantities of sample were dusted onto aluminum sample holders topped with a carbon adhesive tab to provide a suitable background. To mitigate particle charging, all samples were first coated with a ~10 nm layer of gold-palladium nanoparticles using a vacuum sputter coater (Hummer 6.2, Anatech Inc., Richmond, BC, Canada). The morphology of all spray-dried formulations at initial and two months timepoints was assessed from the obtained micrographs with special attention paid to sphericity and any morphological changes such as interparticle agglomeration or the appearance of fibers. 

#### 2.2.9. Raman Spectroscopy

Mannitol polymorphs were identified and analyzed semi-quantitatively using a custom macro-Raman system with a limit of detection of less than 0.5% [28]. The as-received D-Mannitol and Parteck Delta M were measured, with the results providing reference spectra for β-Mannitol and δ-Mannitol, respectively. The α reference spectrum was obtained from a previous study [28]. Spray-dried mannitol-only controls were also analyzed to help isolate the impact of leucine on mannitol polymorphs. In the controls, the concentration of mannitol was the same as in the leucine-containing comparator formulation to provide the same drying kinetics for mannitol in both cases. Spectra were deconvoluted using a non-negative least squares algorithm and normalized intensity factors for the components were calculated with a ±5% certainty. 

#### 2.2.10. X-ray Photoelectron Spectroscopy (XPS)

Surface characterization spectra of select samples were taken on an X-ray photoelectron spectrometer (ESCALAB 250, ThermoScientific, Ottawa, ON, Canada) equipped with a monochromatized aluminum X-ray source with a 500 µm slit size and sampling depth of 8–10 nm. Binding energy scales were adjusted in the spectra plots to ensure that hydrocarbon C1s moieties appear at 284.8 eV. Samples were mounted on indium foil and three measurement sites were analyzed on each sample. The N1s peak at a corrected binding energy of 401.4 eV was used to quantify nitrogen content at the particle’s surface. This surface nitrogen content was compared to the mass fraction of nitrogen in leucine (10.7 wt.%) to determine leucine surface enrichment (as mannitol contains no nitrogen). Error was determined using the standard deviation of three measurements and normalized to the nitrogen content in leucine.

As it was not feasible to characterize all 100 samples by XPS, a relevant subset of the formulations was selected. First, the surface coverage of leucine in aqueous formulations with increasing leucine mass fraction was determined. Additional formulations were selected to expand the understanding of how the ratio of solvents at the found transition region affected the surface concentration of the excipients. A subset of 60 °C formulations manufactured at 40 °C were also selected to represent how changes in outlet temperature affect surface concentrations. The selected formulations are indicated in Table 1.

#### 2.2.11. Karl Fischer (KF) Titration

A coulometric oven titrator (Metrohm 851 Titrando KF, Metrohm USA Inc., Tampa, FL, USA) equipped with a diaphragm-less mode generator electrode was used to determine the water content of select powders. After spray drying, samples with a mass of 80 to 100 mg were hermetically sealed in a crimped KF vial. Drift was corrected with Hydranal water standards and blanks. Samples were preheated to 105 °C before titration.

#### 2.2.12. Gas Chromatography (GC)

The amount of residual ethanol of select spray-dried powders was quantified using a GC system (7890B GC System, Agilent, Santa Clara, CA, USA) equipped with a flame ionization detector and automated headspace sampler. Sample masses from 80 to 100 mg were hermetically sealed in a crimped GC vial and 4.0 mL of dimethylacetamide was injected through the septum. Vials were allowed to equilibrate at 105 °C prior to analysis.

#### 2.2.13. Stability Analysis

The solid-state, visual morphology, and aerodynamic particle size distribution of all spray-dried formulations were screened for a 40 °C and 75% RH stress condition for a two-month timepoint. The methodology used for storing the solid powders to minimize moisture exposure included placing formulations into 60 mL screw-capped HDPE bottles (Cat. No. 2089-0002, Thermo Scientific, Ottawa, ON, Canada). Each bottle was then placed in an aluminum bag (Prod. No. 1390312, Ted Pella Inc., Redding, CA, USA) with a 1-g silica gel desiccant canister. This bag was then heat-sealed. Only PXRD, APS, and SEM measurements were conducted on the two-month sample pulls.

#### 2.2.14. Data Visualization

All contour plots were made using piecewise linear interpolation software (JMP^®^, 16.1.0., SAS Institute Inc., Cary, NC, USA, 1989–2023).

## 3. Results and Discussion

### 3.1. Prediction of Crystallization Windows

The particle formation model was used in to predict the crystallization kinetics of both mannitol and leucine in the spray-dried formulations based on the theoretical critical supersaturation concentrations defined in the Methods section. The model was run for each formulation using the average of the inlet and outlet temperatures as the drying temperature of the droplets, and the times at which critical surface concentrations were reached were determined. These values were then subtracted from each droplet lifetime in order to estimate the available times for crystallization, i.e., the crystallization windows, for leucine and mannitol, as shown in Table 2.

Note that the crystallization windows are the same for the mannitol-only controls and their corresponding binary formulations, as the model does not account for possible interactions between the excipients.

Furthermore, in order to estimate the volume equivalent diameter of the dry particles, the diameter at which the faster component reached its critical supersaturation concentration is also presented in Table 2.

### 3.2. Particle Size

As discussed in the Introduction section, the primary particle size distribution of inhalation powders is an important parameter for successful delivery to the patient. Maintaining a respirable particle size during processing and throughout the shelf life is a key quality attribute. 

#### 3.2.1. Aerodynamic Size Distribution

**Figure 5:**
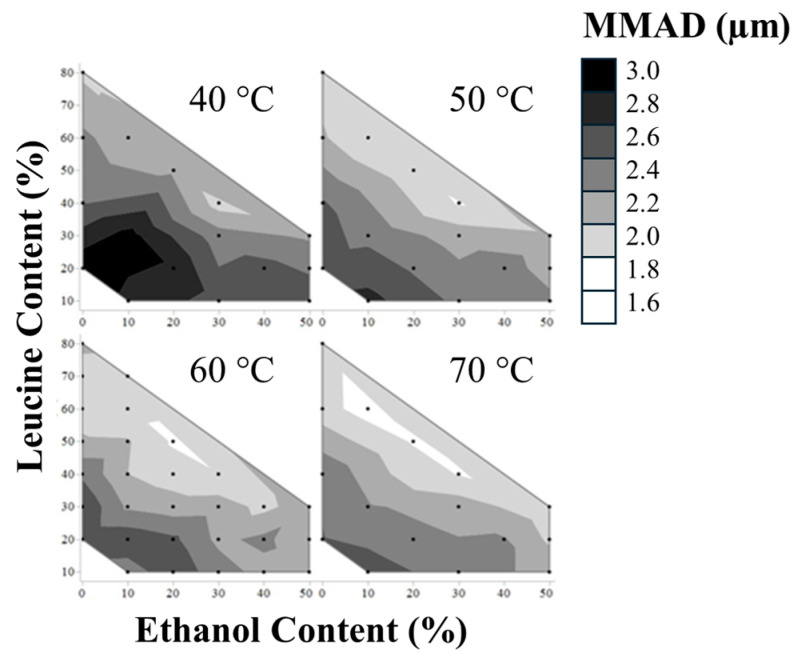
MMADs of all spray-dried formulations measured via an APS instrument.

Aerodynamic diameter distributions of the dispersed powders were assessed via an APS. The resulting MMADs of the formulations are shown in Figure 5.

**Figure 6:**
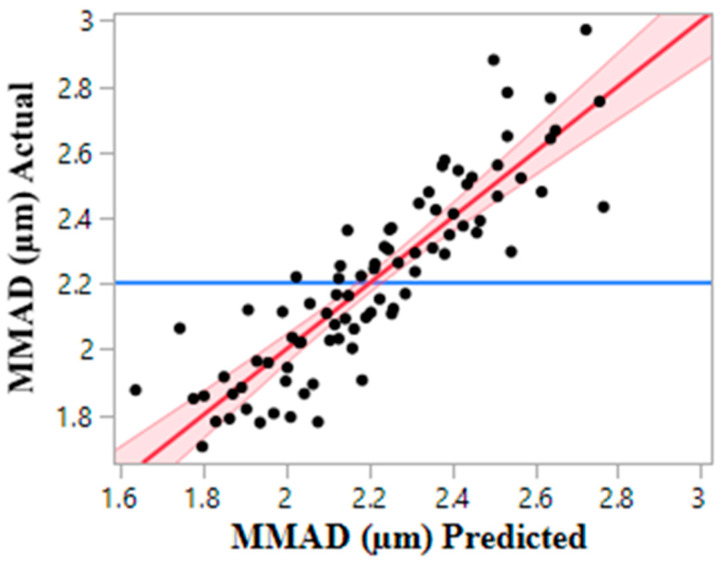
Fit of linear regression of MMADs by plotting predicted vs. actual MMAD values. The red line represents the predicted equation for MMAD (MMAD (µm) = −0.013 (Leucine%) − 0.010 (EtOH%) − 0.009 (Outlet Temperature) + 3.4, *p* value ≤ 0.0001), the red shaded area is the 95% confidence region of the fit, and the blue line is the mean of all MMADs (null hypothesis).

All formulations tested produced particle sizes in the respirable range. It was observed that aerodynamically smaller primary particles or less cohesive powder were produced by an increase in leucine content, ethanol content, and outlet temperature, ordered by decreasing impact. The impact of the parameters was assessed via a linear regression formed by utilizing the logWorth statistic, which is useful when interpreting the degree of difference in importance between a model’s effects. Correlation between experimental data and predicted values is shown in Figure 6. 

It is instructive to compare the measured aerodynamic diameters with theoretical predictions. Assuming a completely dispersed aerosol, mass conservation in combination with the definition of the aerodynamic diameter yields the relationship [29]

(1) $$d_{a}=\sqrt[{6}]{\frac{\rho_{P}}{\rho^{*}}}\sqrt[{3}]{\frac{c_{F}}{\rho^{*}}}d_{0}$$

Here, *d*_a_ is the median aerodynamic diameter and $\rho^{*}$ is the reference density of 1 kg/L. Known parameters are the median initial droplet diameter, *d*_0_, (7.5 µm) and the feed concentration, $c_{F}$, which was 0.02 kg/L for all formulations. Equation (1) shows that the observed differences in aerodynamic diameter must be caused either by incomplete dispersion or by differences in the particle density, *ρ*_P_. The particle density is unknown, but the upper and lower limits can be calculated using the relationship:

(2) $$\rho_{P}{=\rho }_{T}\cdot \left(1-\phi \right)=\frac{\left(1-\phi \right)}{\frac{Y_{\mathrm{Leu}}}{\rho_{\mathrm{Leu}}}+\frac{Y_{\mathrm{Man}}}{\rho_{\mathrm{Man}}}}$$

The particle density is lower than the average true density of the particle, *ρ*_T_, if the particle contains void space, e.g., for a hollow particle. *ϕ* denotes the ratio of void volume to the total volume of the particle. The true density of the particle material is a function of the mass fractions for leucine and mannitol, *Y*_Leu_ and *Y*_Man_, and the true densities of the excipients, *ρ*_Leu_ (1.29 kg/L) [26] and *ρ*_Man_ (1.51 kg/L) [30]. The highest possible particle density, which is reached for a pure mannitol particle without void space, yields an aerodynamic diameter of 2.2 µm. Many measured values exceed this calculated maximum. Therefore, we can conclude that either the powder was not completely dispersed in some formulations or that irreversible aggregation or fusing of particles had occurred. 

The smallest measured MMAD, 1.7 µm (formulation L60Et10T70), agrees with the corresponding calculated result for a completely dispersed powder and a void space of approximately 50%, which is a realistic value considering the associated hollow particle morphologies observed here. This indicates that the powders with low aerodynamic diameters were also easier to disperse, i.e., less cohesive.

#### 3.2.2. Optical Particle Size Distribution

**Figure 7:**
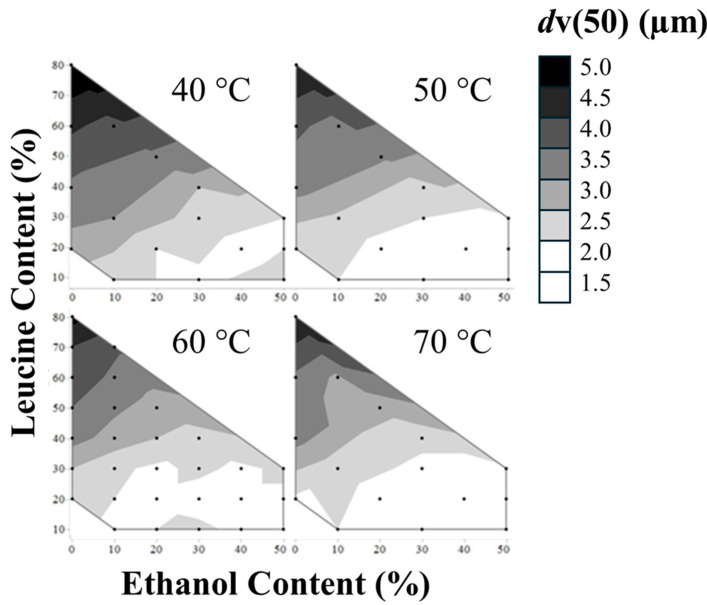
*d*v(50)s of all formulations measured at an initial timepoint (t0).

All optical particle size distributions were collected using a Malvern Mastersizer 3000 with dry powder disperser and analyzed using Mie theory. The volume distribution summary statistic *d*_v_(50), is reported in Figure 7 as a function of process and formulation variables. Utilizing the logWorth statistic to determine impact led to the use of a full factorial model with only leucine and ethanol content having a statistically significant impact.

**Figure 8:**
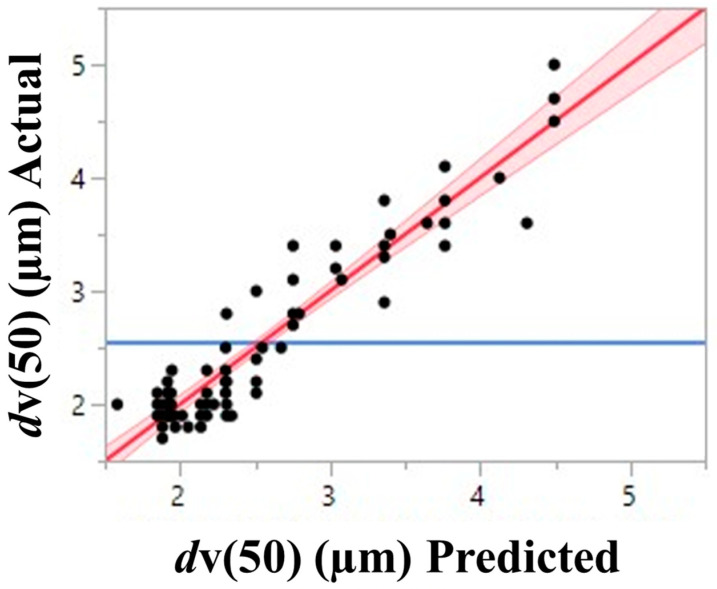
Fit of full factorial of *d*v(50) by plotting predicted vs. actual *d*v(50). The red line represents the predicted equation for *d*v(50) (alternative hypothesis), the red shaded area is the 95% confidence region of the fit, and the blue line is the mean of *d*v(50)s (null hypothesis).

According to the least squares regression model shown in Figure 8, leucine and ethanol content have a similar yet opposite impact on *d*v(50). Conversely, from the trends seen in the MMAD, an increase in the leucine content led to an observable increase in *d*v(50). Whereas, similarly to MMAD, an increase in ethanol content leads to a decrease in *d*v(50). Outlet temperature does not play a statistically significant role in *d*v(50). 

The equivalent optical diameter has sometimes been interpreted as a volume equivalent diameter. However, in the case of non-spherical, inhomogeneous particles (see Figure 8), this is not a valid assumption as can be seen by a simple theoretical calculation of the volume equivalent diameter, *d*_V_ [31]:

(3) $$d_{v}=\sqrt[{3}]{\frac{c_{F}}{\rho_{P}}}d_{0}$$

For the smallest measured equivalent optical diameter of 1.5 µm, a particle density of 2.5 kg/L would have to be used in Equation (3) to reach a similar volume equivalent diameter. This exceeds the maximum possible particle density by a large margin, indicating that the results of optical particle sizing should not be interpreted as volume equivalent diameters for these types of particles.

**Figure 9:**
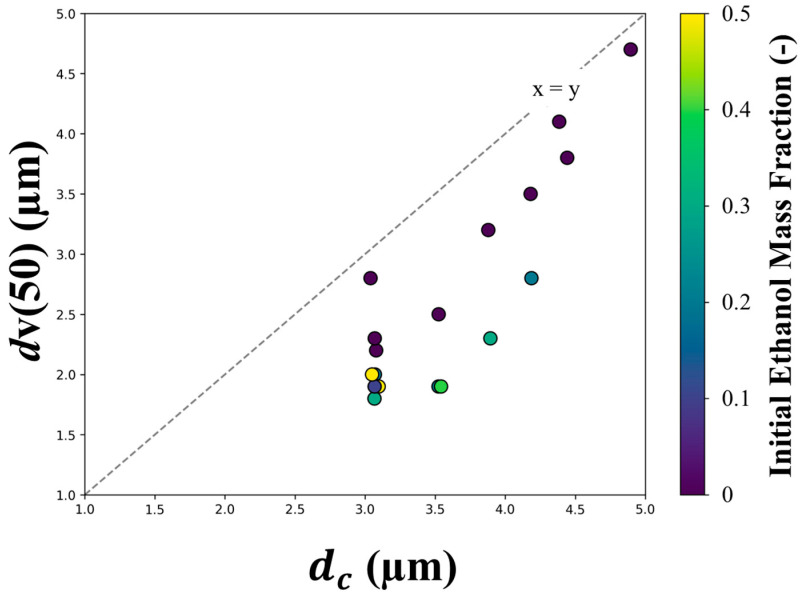
The median optical diameter of the spray-dried particles, *d*v(50), compared to the predicted diameter at which the first component reaches its critical supersaturation. The color coding denotes the initial ethanol mass fraction.

The measured optical particle sizes, *d*v(50), were also compared to the predicted diameter at which the first component reached critical supersaturation, $d_{c}$, in Figure 9. Large equivalent optical diameters are expected from particles with a large volume equivalent diameter, i.e., a low particle density. Particles with low particle density are formed when shell formation occurs early in the droplet evaporation, arresting droplet shrinkage when the droplet diameter is still large. The exact point in time—and the corresponding droplet diameter—when a rigid shell is formed on the droplet cannot be calculated with the current particle model, but we know that it cannot happen earlier than the onset of nucleation, which is predicted to occur when the droplet surface reaches critical supersaturation. Furthermore, the shell subsequently tends to deform or collapse affecting the measured optical diameter. Hence, the final dry particle is expected to have a diameter that correlates with but is lower than $d_{c}$, which is in fact observed in Figure 9. It should also be mentioned that formulations with higher initial ethanol fraction had reduced surface tension of the feed, resulting in marginally smaller initial droplets. This effect is not accounted for in the theoretical predictions as the initial droplet diameter was assumed to be constant among all formulations. This may explain in part why the points representing higher ethanol concentrations are farther away from the identity line.

### 3.3. Solid State and Chemical Composition

In order to better understand the co-nucleation kinetics of leucine and mannitol, several orthogonal techniques were used to qualitatively and quantitatively determine mannitol polymorphism and leucine surface enrichment.

#### 3.3.1. Powder X-ray Diffraction (PXRD)

**Figure 10:**
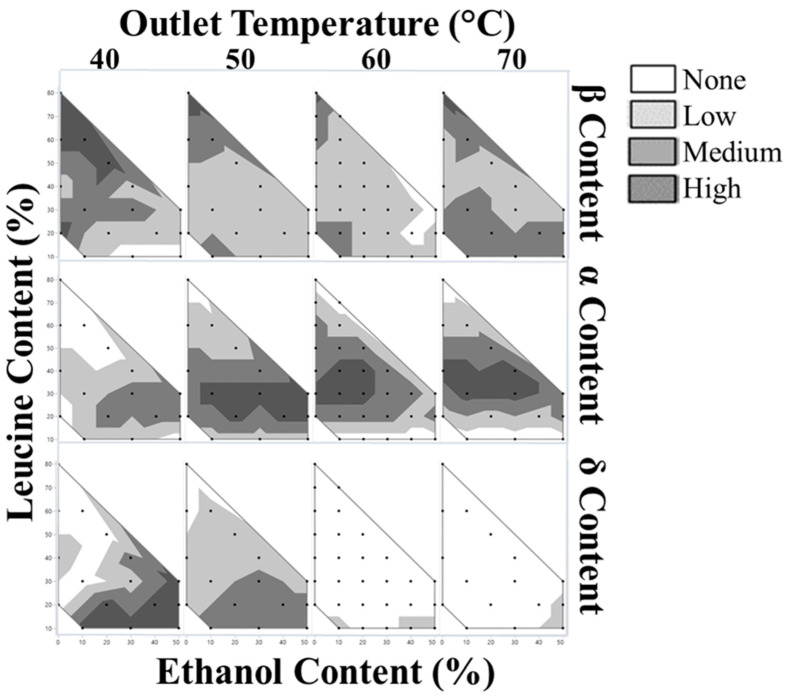
Qualitative intensity of mannitol polymorphs via PXRD in order of descending stability; β-mannitol (**top**), α-mannitol (**middle**), and δ-mannitol (**bottom**).

All formulations were initially screened for mannitol polymorphism and overall crystallinity using a powder X-ray diffractometer. The resulting diffractograms showed that all samples were fully crystalline within the limit of detection with no amorphous halo present. Known characteristic peaks for mannitol polymorphs were used to determine the qualitative intensity of each polymorph in the samples. The diffractograms were inspected for peaks at 6.2°, 9.6°, 14.6°, and 17.2° for leucine, δ, β, and α-mannitol, respectively. The qualitative content of each polymorph for all samples are summarized in Figure 10.

β-mannitol, the most stable and desired form, was present in all formulations except four: L10Et30T40, L10Et50T40, L20Et40T60, and L30Et50T60. These formulations all have lower relative leucine loadings and a higher content of ethanol. The α-mannitol content appeared stratified, with the highest qualitative content appearing between 20–40 wt.% leucine for all outlet temperatures and ethanol contents. The δ-mannitol qualitative content increased with decreasing outlet temperatures, with higher content at higher concentrations of ethanol. Outlet temperatures of 60 and 70 °C largely showed no δ peak. 

#### 3.3.2. Raman Spectroscopy

In order to corroborate the PXRD data with a high-sensitivity technique that has been shown to be able to deconvolute the polymorphs of mannitol [29], a customized macro-Raman system was utilized. In addition to selected mannitol/leucine formulations, mannitol-only controls were spray dried at an identical mannitol concentration to their corresponding binary formulation. This was used to isolate the impact of leucine in mannitol polymorphism. The collected spectra showed that all formulations were completely crystalline within the limit of detection.

**Figure 11:**
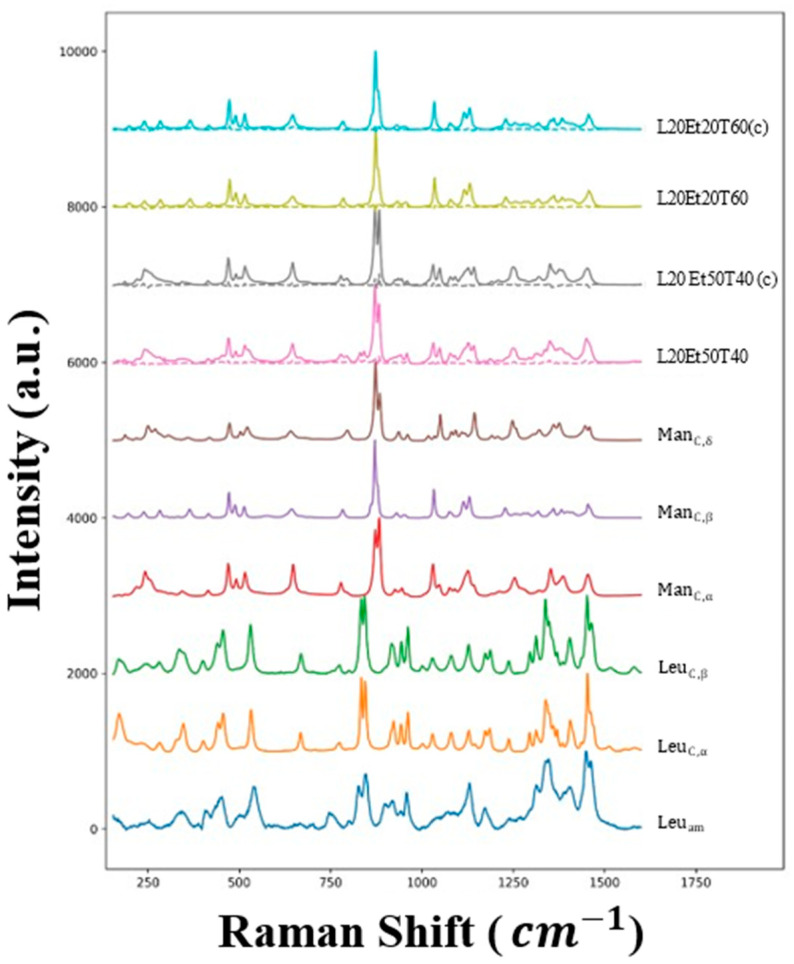
The raw (closed lines) and residual (dashed lines) Raman spectra of some of the mannitol-only control and binary formulations, as well as the reference spectra used for the deconvolution. C and am refer to crystalline and amorphous phases, respectively, while (c) refers to the mannitol-only control formulations.

When comparing formulations with different mannitol/leucine ratios, the mannitol concentration in the spray solution also changed. As both mannitol and leucine are crystallizing excipients, it was thought that the surface competition of the excipients may play a significant role in mannitol polymorphism. To determine if this is the case, the mannitol-only spray’s polymorphic forms were analyzed using Raman spectroscopy and compared to their corresponding binary formulations. The Raman spectra of selected mannitol control powders, their corresponding binary counterparts and their residual spectra after deconvolution, as well as the reference spectra are shown in Figure 11.

A subset of the binary formulations was selected for Raman analysis to ensure adequate coverage of all desired test parameters and correlated with PXRD results. Normalized δ-mannitol to total mannitol content was calculated based on the relative polymorphic intensities found via the deconvolution. Formulations with δ-mannitol content are shown in Table 3. Upon spectra deconvolution, all of the mannitol control samples were in the most thermodynamically stable form, β-mannitol except L20Et50T40(c), which showed some δ- and α-mannitol intensities. Of the formulations tested, there were no statistically-significant differences in polymorphism between mannitol-only controls and leucine-mannitol paired samples. It appears that leucine does not interfere with mannitol polymorphism.

The data do suggest, however, that the processing conditions and co-solvent composition do effect mannitol polymorphism, specifically that high ethanol concentration and low outlet temperatures can result in a less-stable polymorph’s nucleation during drying. All of the formulations tested that were manufactured at an outlet temperature of 40 °C contained δ-mannitol. Of those formulations, the ones with the highest ethanol content, L20Et50T40 and L30Et40T40, had the highest δ-mannitol content. The only formulation manufactured at 60 °C that contained δ-mannitol, L10Et20T60, had neither high ethanol content nor leucine content, suggesting that the nucleation kinetics of this system are affected by temperature. With this, the trends in mannitol polymorphism observed at a 40 °C outlet temperature are not able to be extrapolated to other outlet temperatures studied.

#### 3.3.3. X-ray Photoelectric Spectroscopy (XPS)

**Figure 12:**
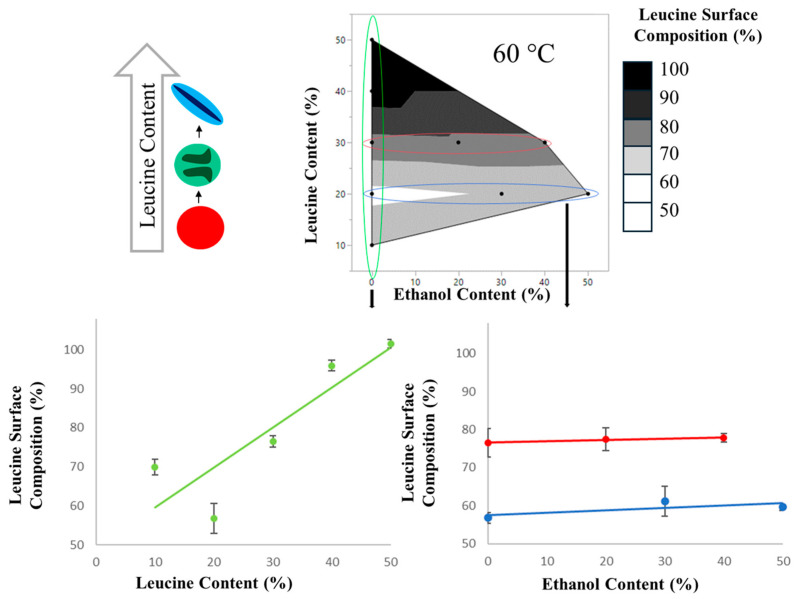
Graphical representation of leucine surface composition. Contour plot (**Top**) shows data collected on 60 °C outlet samples. The scatter plots (**Bottom**) show in greater detail the resulting trends in leucine surface composition with respect to ethanol content (Right, Red line for 20% leucine, Blue line for 20% leucine), and leucine content (Left, green line for 0% ethanol)).

In order to quantitatively determine the surface enrichment of the particles using atomic percentages, an X-ray photoelectric spectrometer was utilized. The resulting leucine surface composition is shown in Figure 12. The N1s peak and the amide C1s peak were present in all samples, indicating that some leucine was on the surface of all particles. Even the formulation with the lowest tested leucine loading, L10Et0T60, showed that the surface composition of the particles was over 50% leucine. As shown in Table 4, the same formulations manufactured at 40 °C consistently had higher leucine surface concentrations than their 60 °C counterparts. This correlates with the theoretical predictions, which showed that at lower temperatures, leucine has a larger window of crystallization. This allows the much less soluble leucine to cover the surface of the particles. Additionally, ethanol content appears to play a minor role in leucine surface composition; a minor decrease in leucine surface composition trends with an increase in ethanol content. The highest leucine loading resulted in the highest leucine surface enrichment. This correlates with the assumption that if there is more leucine available to nucleate on the particles’ surface, greater surface enrichment is achieved.

XPS was the only technique utilized that could quantitatively determine the extent of each excipient on the surface. From the XPS data, it was revealed that leucine was surface-enriched in all tested samples, relative to the composition of the bulk particle. This included the sample with the lowest leucine loading, 10 wt.% leucine (L10Et0T60). Only the sample with 50 wt.% leucine was fully surface-enriched, with a surface concentration of ~100%. This indicates that leucine enrichment occurred at all leucine loadings studied, however early leucine shell formation was required for complete surface cover. Moreover, it was found that changes in SEM morphology occurred only when the leucine surface concentration exceeded 75%.

**Figure 13:**
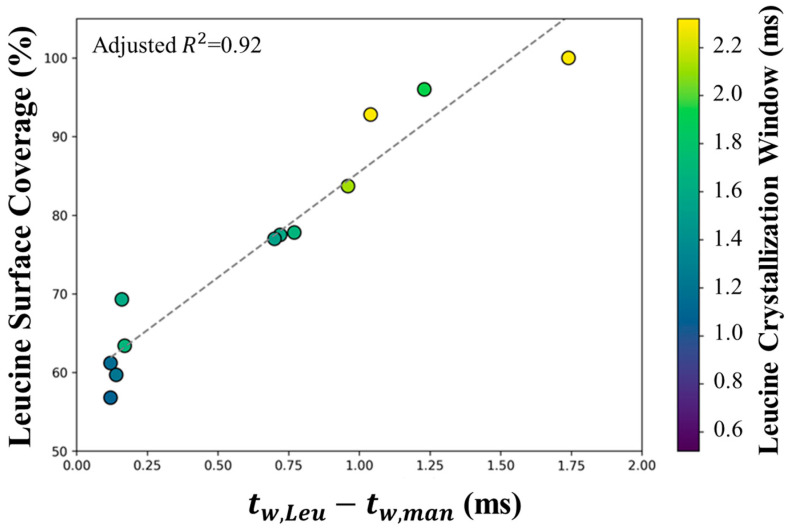
The leucine surface coverage from XPS measurements compared to the theoretically estimated difference in available time for crystallization of leucine and mannitol during spray drying. The color coding denotes the crystallization window of leucine.

In order to explain the different levels of surface coverage and find a trend in these complex spray-dried systems, the XPS measurements were compared to the crystallization windows of leucine and mannitol obtained from the mathematical model as shown in Figure 13. A clear relationship is visible between the difference in the crystallization windows of leucine and mannitol and leucine surface coverage. This difference indicates the sequence of nucleation and how much earlier leucine nucleates compared to mannitol. In all cases analyzed by XPS, leucine is predicted to nucleate first, but the amount of surface coverage increases the earlier leucine crystallizes relative to mannitol. This shows the importance of the kinetics of nucleation and crystal growth of leucine and mannitol, because both excipients crystallize and thus compete for accumulation on the surface of the evaporating droplets. It is also seen from this plot that even when leucine and mannitol nucleated at similar times, high leucine surface coverages of more than 50% were obtained. A possible explanation is the surface activity of leucine, which has been predicted to lead to the rapid formation of a thin leucine layer at the surface [12]. For the samples studied, 0.7 ms longer crystallization time was required compared with mannitol to achieve at least 75% leucine surface enrichment.

### 3.4. Particle Morphology

Particle morphology plays a significant role in a drug products’ dispersibility from a dry powder inhaler. The morphology of the particle can dictate whether particles easily de-agglomerate upon actuation from an inhalation device. Particles with a collapsed shell morphology have less contact area with other particles versus spherical particles [32]. This diminished contact area can allow particles to deagglomerate readily and maintain adequate respirable size. With a higher particle-to-particle contact area, breath-actuated dispersive forces must be strong enough to then overcome the inter-particle forces [32]. Shell collapse has also been cited as a characteristic sign of adequate leucine surface concentration [8].

#### Scanning Electron Microscopy (SEM)

**Figure 14:**
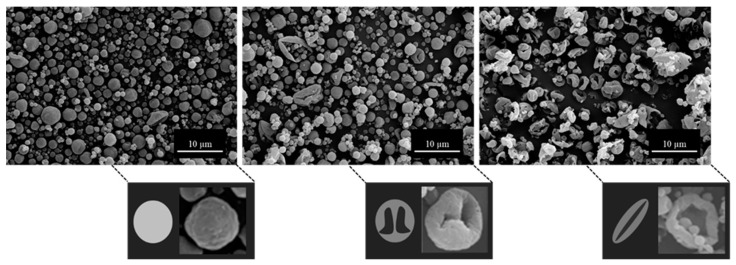
SEM particle morphology categories: spheres (**left**), partially-collapsed (**middle**), and collapsed (**right**). Representative images and cartoons of a single particle are shown as well.

Electron micrographs of all particles were taken at three sites for each sample. As it was not possible to use image scanning software to quantitatively define the extent of particle collapse due to particles overlapping, the bulk morphology of each site was observed and classified visually. Formulations fell into one of three categories; spherical, collapsed, or a transition region of partially-collapsed. A formulation was categorized as having spherical morphology if >90% of the particles were spherical. Similarly, a formulation was categorized as having collapsed morphology if >90% of particles had a major indent leading to the slight elongation of the particles that greatly affected its sphericity. The partially-collapsed morphology was identified due to a need to classify when indentation of the larger particles was present that affected sphericity, but no indentation on the smaller particles was observed. Examples of each classification of bulk particle morphology can be seen in Figure 14.

**Figure 15:**
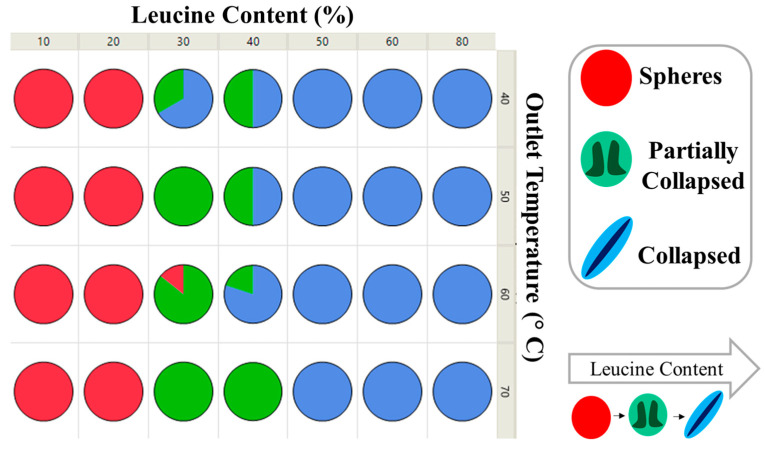
SEM morphology as a function of leucine content and outlet temperature. Each pie graph contains all solvent mixtures tested of formulations with select leucine content and outlet temperature. The color of each pie graph corresponds to a specific morphology: red (spheres), green (partially collapsed), and blue (collapsed).

The initial particle morphology of all formulations was heavily dependent upon which component crystallized first. As leucine content increased, leucine commenced crystallization progressively earlier than mannitol, and the resulting particles became proportionally more collapsed. This can be seen in Figure 15, where formulations are primarily separated by leucine content and secondarily by outlet temperature. The multiple samples represented in each pie graph consist of all tested solvent mixtures at that leucine loading and outlet temperature. Below 20 wt.% leucine, all samples had spherical morphology, regardless of outlet temperature and ethanol content. Compared with the XPS results, these correspond to lower levels of leucine on the surface. Similarly, above 50 wt.% leucine, all samples were fully collapsed and correspond well with XPS samples where leucine was surface dominant. There appears to be a transitional region from 30–40 wt.% leucine, where ethanol and outlet temperature may have some sway over the resulting particle morphology. For instance, L30Et0T60 was spherical, L30Et30T60 was partially collapsed, and L30Et30T40 was collapsed.

**Figure 16:**
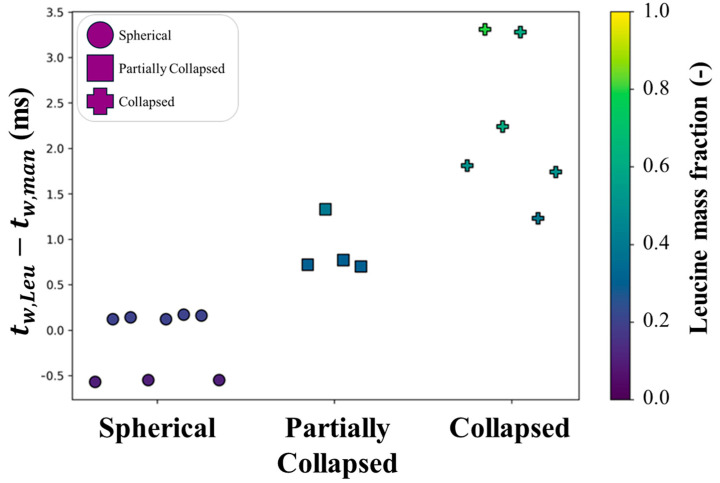
The observed particle shapes as compared to the predicted difference in crystallization windows between leucine and mannitol. The color coding denotes the leucine mass fraction in the feed.

The predicted crystallization sequence of leucine and mannitol was also used to explain the different morphologies observed, as shown in Figure 16. For the samples studied, an earlier predicted time of crystallization for leucine resulted in collapsed particles, compared to the generally spherical particles for which mannitol was expected to have similar times available for crystallization, i.e., nucleate and crystallize together with leucine. To achieve the desired morphology in this system, leucine needs to be given enough time to crystallize and be allowed to crystallize earlier than the competing crystallizing excipient, mannitol.

### 3.5. Residual Solvent

Moisture content and residual solvent from processing can impact physical stability of inhaled formulations. A subset of samples was tested for residual water and ethanol content. All formulations tested via KF had less than 1 wt.% water content post spray drying. Residual water content inversely trends with leucine content instead of the predicted factor of ethanol/water ratio. All formulations tested via GC had less than 0.3 wt.% ethanol content post spray drying. Residual ethanol content proportionally trends with an increase in ethanol in the initial solvent mixture as expected. Residual ethanol content was not correlated with leucine content.

### 3.6. Stability

One major benefit of dry powder inhalation formulations is their potential for improved room-temperature stability over liquid formulations. A two-month accelerated stability study was conducted on samples stored at 40 °C and 75% humidity. Powders were packaged in HDPE bottles within heat-sealed mylar bags that contained desiccant to mimic common packing conditions. All formulations were analyzed post-stability via APS, PXRD, and SEM to study the products’ aerosol properties, polymorphism, and visual morphology.

To test that particles are still able to deagglomerate post-stability and remain aerodynamically relevant, the MMAD was measured again. The MMAD of all formulations remained within ±0.5 µm of the initial value and all were still respirable.

**Figure 17:**
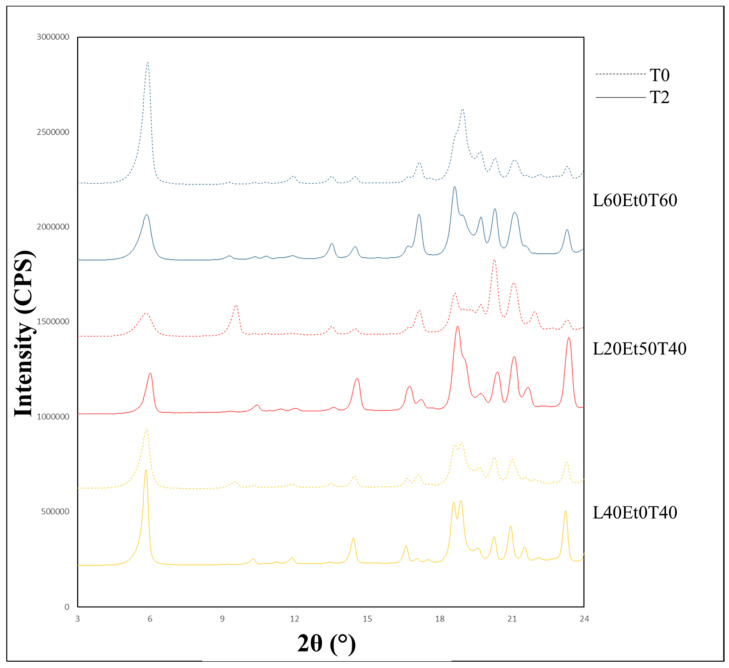
Diffractograms of powders whose mannitol polymorphs interconverted during stability, comparing initial (dashed) and two-month timepoints (solid).

Utilizing PXRD post stability is an accessible way of determining if polymorph interconversion occurred during two months at 40 °C. Diffractograms pre- and post-stability were overlaid to determine if either a clear change in peak ratios, or the loss or addition of a peak had occurred. Only three tested formulations had any of these discrepancies; L20Et50T40, L40Et0T40, and L60Et0T60, which are shown in Figure 17. Both L20Et50T40 and L40Et0T40 showed a loss of the characteristic δ-peak at 9.6° in favor of interconverting to the more stable β polymorph. L60Et0T60 was the only formulation that showed a stark polymorphic interconversion from α-mannitol to β-mannitol. All other formulations had the same relative intensity in all peaks, or had all peaks in the diffractogram shifted, all of which could be accounted for in minute differences in sample preparation. As expected, polymorphic interconversion only occurred from less stable polymorphs to the most stable polymorph, β-mannitol.

**Figure 18:**
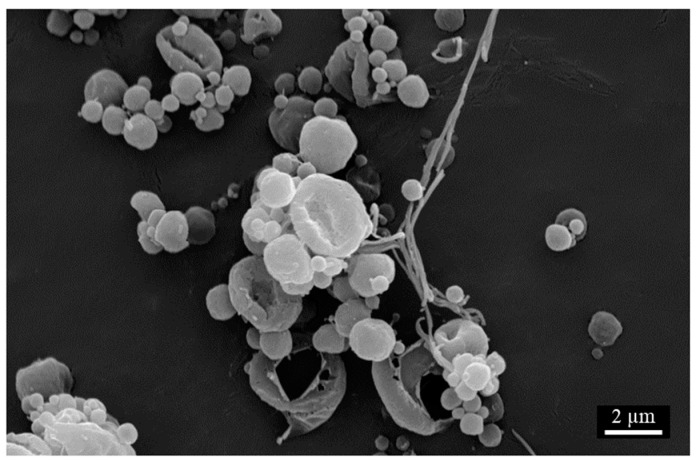
Representative image of fibers observed on two-month stability.

SEM was used to assess the impact of storage on particle morphology. In addition to spherical and collapsed particles, the formation of some fibers was observed at the two-month time point. From sample to sample, these fibers ranged in severity, size, and amount per particle, and an example is shown in Figure 18. These fibers were previously reported and shown to not impact powder performance [10].

## 4. Conclusions

This work studied the interplay of process and formulation parameters for inhalable dry powder leucine-mannitol formulations, in which both components crystallize during spray drying. The spray-dried formulations demonstrated here probed the impact of formulation composition, the ethanol/water ratio in the spray solvent, and outlet temperature on a particle’s surface composition, morphology, and polymorphic state. Advanced particle formation models helped quantify the competition for the surface between the two crystallizers. For this system, it was shown that there was no detectable interaction between leucine and the polymorphic form of mannitol, the crystalline model active. With this, it was shown that the advantages of leucine, i.e., lower particle density and low hygroscopicity, can be achieved even for formulations heavily constrained by leucine’s low solubility in the co-solvent system. A sufficiently-high leucine crystallization window and a crystallization sequence that allows leucine to crystallize first is necessary to achieve sufficient enrichment of leucine at the surface, which then leads to the desired collapsed particle morphology. The results of this study indicate that formulation with leucine is feasible and likely beneficial for inhalable dry powders, even when the formulation is fully crystalline and the active is poorly soluble in water. This finding should be verified with other crystallizing actives. Formulation and process design in these systems profits from mechanistic understanding of the particle formation process and the application of corresponding predictive models.

## Figures and Tables

**Table 1:** All formulations explored during the study (*n* = 88). Lxx represents the percentage of leucine in the formulation, Etxx represents the percentage of ethanol in the solvent mixture. ‘x’ denotes XPS surface analysis, ‘m’ denotes theoretically modeled conditions, and ‘s’ denotes conditions that were studied, but not theoretically modeled or analyzed via XPS. (c) in the formulation name denotes mannitol-only control.

Formulation Name	Feed Concentration (%)			Outlet Temperature (Txx)			
LxxEtxx	Ethanol: Water	Leucine	Mannitol	40 °C	50 °C	60 °C	70 °C
L10Et0	0:100	0.2	1.8			m,x	
L20Et0	0:100	0.4	1.6	m,x	s	m,x	s
L20Et0(c)	0:100	0	1.6	m		m	
L30Et0	0:100	0.6	1.4			m,x	
L40Et0	0:100	0.8	1.2	s	s	m,x	s
L50Et0	0:100	1.0	1.0			m,x	
L60Et0	0:100	1.2	0.8	m	s	m	s
L70Et0	0:100	1.4	0.6			s	
L80Et0	0:100	1.6	0.4	s	s	m	s
L80Et0(c)	0:100	0	0.4			m	
L10Et10	10:90	0.2	1.8	s	s	m	s
L20Et10	10:90	0.4	1.6			s	
L30Et10	10:90	0.6	1.4	s	s	s	s
L40Et10	10:90	0.8	1.2			s	
L50Et10	10:90	1.0	1.0			s	
L60Et10	10:90	1.2	0.8	s	s	s	s
L70Et10	10:90	1.4	0.6			s	
L10Et20	20:80	0.2	1.8			m	
L10Et20(c)	20:80	0	1.8			m	
L20Et20	20:80	0.4	1.6	s	s	s	s
L30Et20	20:80	0.6	1.4	m,x *		m,x	
L40Et20	20:80	0.8	1.2			s	
L50Et20	20:80	1.0	1.0	s	s	m	s
L50Et20(c)	20:80	0	1.0			m	
L10Et30	30:70	0.2	1.8	s	s	s	s
L20Et30	30:70	0.4	1.6			m,x	
L20Et30(c)	30:70	0	1.6			m	
L30Et30	30:70	0.6	1.4	s	s	s	s
L40Et30	30:70	0.8	1.2	s	s	m	s
L10Et40	40:60	0.2	1.8			s	
L20Et40	40:60	0.4	1.6	s	s	s	s
L30Et40	40:60	0.6	1.4	m,x *		m,x	
L10Et50	50:50	0.2	1.8	s	s	s	s
L20Et50	50:50	0.4	1.6	m,x	s	m,x	s
L20Et50(c)	50:50	0	1.6	m		m	
L30Et50	50:50	0.6	1.4	s	s	s	s

* Indicates formulations that were not analyzed via PXRD or SEM.

**Table 2:** The predicted crystallization windows of leucine and mannitol as well as the diameter at which the faster component reached critical supersaturation. The bolded entries indicate which excipient nucleates first.

Formulation	$t_{w,leu}$ (ms)	$t_{w,man}$ (ms)	$d_{c}$ (μm)
L10Et0T60	0.52	**1.07**	3.07
L20Et0T60	**1.08**	0.96	3.08
L30Et0T60	**1.53**	0.83	3.52
L40Et0T60	**1.94**	0.71	3.88
L50Et0T60	**2.32**	0.58	4.18
L60Et0T60	**2.66**	0.42	4.44
L80Et0T60	**3.32**	0.01	4.89
L10Et10T60	0.53	**1.08**	3.07
L10Et20T60	0.54	**1.11**	3.07
L30Et20T60	**1.58**	0.86	3.52
L50Et20T60	**2.4**	0.59	4.19
L20Et30T60	**1.14**	1.02	3.07
L40Et30T60	**2.08**	0.75	3.89
L30Et40T60	**1.68**	0.91	3.54
L20Et50T60	**1.21**	1.07	3.10
L20Et0T40	**1.6**	1.44	3.04
L60Et0T40	**3.94**	0.66	4.38
L30Et20T40	**2.32**	1.28	3.48
L30Et40T40	**2.12**	1.16	3.51
L20Et50T40	**1.69**	1.52	3.05

**Table 3:** Summary of Raman measurements of δ content in binary formulations and the corresponding mannitol control. Formulations are ordered from lowest δ content to highest.

Formulation Name	Normalized δ Content	Mannitol Control	Normalized δ Content
LxxEtxxTxx	(%)	LxxEtxxTxx(c)	(%)
L20Et0T60	0	L20Et0(c)	0
L60Et0T60	0	-	-
L80Et0T60	0	L80Et0(c)	0
L10Et10T60	0	-	-
L10Et20T60	1 ± 5	L10Et20(c)	0
L30Et20T60	0	-	-
L50Et20T60	0	L50Et20(c)	0
L20Et30T60	0	L20Et30(c)	0
L40Et30T60	0	-	-
L30Et40T60	0	-	-
L20Et50T60	0	L20Et50(c)	0
L20Et0T40	1 ± 5	L20Et0(c)	0
L60Et0T40	10 ± 8	-	-
L30Et20T40	0	-	-
L30Et40T40	20 ± 6	-	-
L20Et50T40	40 ± 5	L20Et50(c)	45 ± 5

**Table 4:** Formulations explored via XPS and the resulting leucine surface composition. The table is separated by outlet temperature (60 °C left; 40 °C right).

Binary Formulation	Leucine Surface Composition	Binary Formulation	Leucine Surface Composition
LxxEtxxT60	(%)	LxxEtxxT40	(%)
L10Et0T60	70 ± 2	-	-
L20Et0T60	57 ± 4	L20Et0T40	70 ± 2
L30Et0T60	77 ± 2	-	-
L40Et0T60	96 ± 2	-	-
L50Et0T60	102 ± 2	-	-
L30Et20T60	78 ± 1	L30Et20T40	93 ± 3
L20Et30T60	61 ± 3	-	-
L30Et40T60	78 ± 1	L30Et40T40	84 ± 2
L20Et50T60	60 ± 1	L20Et50T40	63 ± 3

## Data Availability

Data are contained within the article.
